# A ROCK inhibitor suppresses the transforming growth factor-beta-2-induced endothelial–mesenchymal transition in Schlemm’s canal endothelial cells

**DOI:** 10.1038/s41598-023-36808-8

**Published:** 2023-06-14

**Authors:** Tomokazu Fujimoto, Miyuki Inoue-Mochita, Toshihiro Inoue

**Affiliations:** 1 Department of Ophthalmology, Faculty of Life Sciences, Kumamoto University, 1-1-1 Honjo, Chuo-ku, Kumamoto, 860-8556 Japan; 2 Department of Medical Cell Biology, Institute of Molecular Embryology and Genetics, Kumamoto University, Kumamoto, Japan

**Keywords:** Cell biology, Eye diseases, Ocular hypertension

## Abstract

In the normal eye, most of the aqueous humor drains through the trabecular meshwork (TM) and Schlemm’s canal (SC). The concentration of transforming growth factor beta 2 (TGF-β2) is increased in the aqueous humor of primary open angle glaucoma patients. TGF-β2 increases outflow resistance by affecting the TM and SC, and endothelial–mesenchymal transition (EndMT) of SC cells is involved in these changes. Here, we investigated the effect of a ROCK inhibitor on TGF-β2-induced EndMT in SC cells. The ROCK inhibitor Y-27632 suppressed the TGF-β2-induced increase in the trans-endothelial electrical resistance (TER) and proliferation of SC cells. Y-27632 suppressed the expression of α-SMA, N-cadherin, and Snail, which are upregulated by TGF-β2. Moreover, TGF-β2 decreased mRNA levels of bone morphogenetic protein (BMP) 4 and increased those of the BMP antagonist gremlin (GREM1), but Y-27632 significantly suppressed these changes. Y-27632 also inhibited TGF-β2-induced phosphorylation of p-38 mitogen-activated protein kinase (MAPK). BMP4 and the p-38 MAPK inhibitor SB203580 suppressed the TGF-β2-induced TER elevation in SC cells. Moreover, SB203580 suppressed TGF-β2-induced upregulation of fibronectin, Snail, and GREM1. These results indicate that a ROCK inhibitor inhibited the TGF-β2-induced EndMT in SC cells, implying the involvement of p38 MAPK and BMP4 signaling.

## Introduction

Glaucoma is a major cause of blindness^1^, and elevated intraocular pressure is a major risk factor for the progression of glaucoma^2,3^. Intraocular pressure is regulated by the production and draining of aqueous humor in the eye. Aqueous humor is drained through the conventional and the uvea-sclera outflow tracts, mostly via the former^4,5^. The trabecular meshwork (TM) and Schlemm’s canal (SC) endothelium, which constitute the conventional outflow, regulate aqueous humor outflow. In glaucoma patients, decreased TM cells and abnormal extracellular matrix (ECM) deposition in the TM are observed^6,7^, resulting in increased outflow resistance and intraocular pressure. The concentration of transforming growth factor beta 2 (TGF-β2) is increased in aqueous humor of primary open angle glaucoma (POAG) patients^8–11^. TGF-β2 increases aqueous humor outflow resistance, which is thought to be related to epithelial–mesenchymal transition (EMT) induction of TM cells by TGF-β2, such as increased expression of ECM (fibronectin, collagen) and alpha smooth muscle actin (α-SMA)^12–16^. Moreover, TGF-β2 induces the endothelial mesenchymal transition (EndMT) in SC cells^17,18^. In addition, SC cells from glaucoma patients have higher expression of α-SMA, fibronectin, and collagen, and higher proliferative potential than those from non-glaucoma eyes^19,20^. Such changes in SC cells from glaucoma patients are identical to those induced by TGF-β2 in SC cells, implicating EndMT induction of SC cells in the pathogenesis of glaucoma.

Rho kinase inhibitor has been used as a therapeutic agent for glaucoma and has an effect by decreasing intraocular pressure due to a decrease in the resistance of the conventional outflow^21–24^. ROCK inhibitors alter TM cell morphology, suppress extracellular matrix production, enhance resistance to oxidative stress, and promote phagocytosis^23,25–28^. In addition, they increase the permeability of SC cells by increasing giant vacuoles and decreasing cell-to-cell adhesion^29^. Furthermore, they suppress the steroid-induced increase in trans-endothelial electrical resistance (TER) in SC cells and that in ECM expression in TM cells, and ameliorate the steroid-induced increase in aqueous outflow resistance^30^. However, there are no reports on the effects of ROCK inhibitors on the changes in SC cells induced by TGF-β2. We investigated the effects of ROCK inhibitors on TGF-β2-induced EndMT in SC cells.

## Results

### Effect of a ROCK inhibitor on the TGF-β2-induced TER increase and cell proliferation

**Figure 1:**
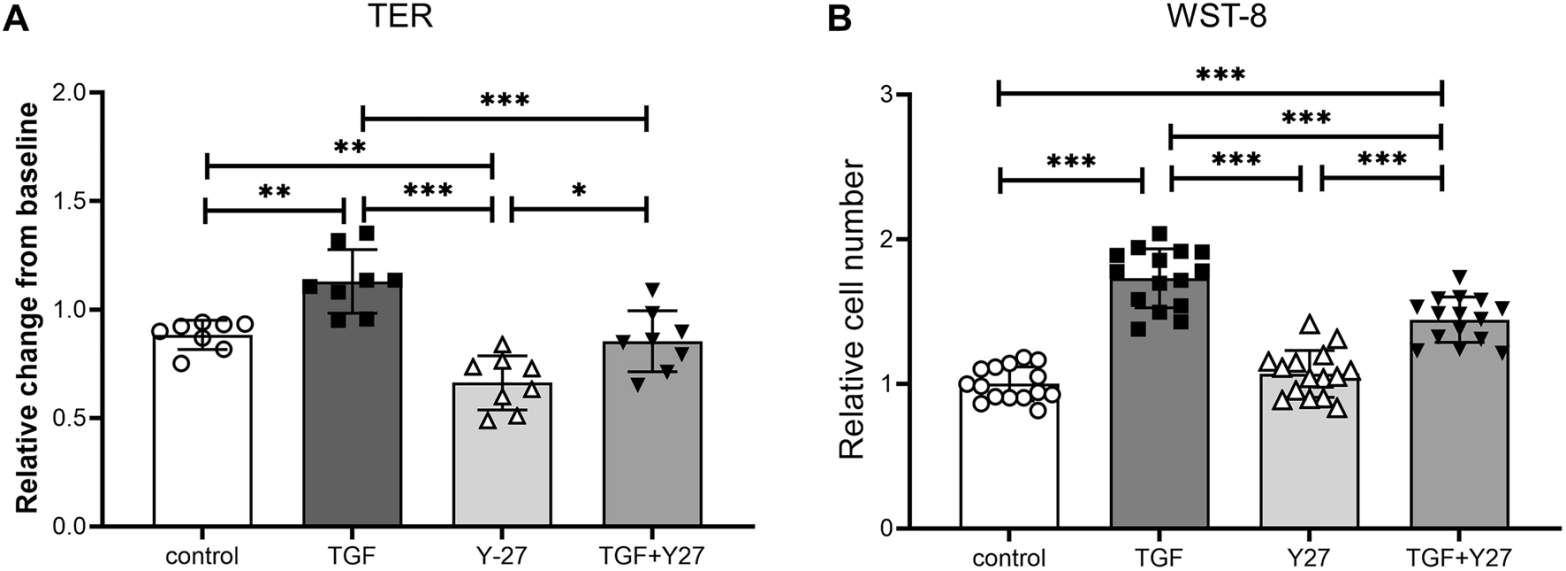
Effects of TGF-β2 and Y-27632 on trans-endothelial electrical resistance (TER) and proliferation in monkey Schlemm’s canal (SC) endothelial cells. SC cells were treated with 5 ng/mL TGF-β2 and/or 10 µM Y-27632 for 72 h. (**A**) TER values are shown as relative changes from baseline. (**B**) Proliferation of SC cells evaluated using WST-8 assay. Data are means ± SD; n = 8 for TER; n = 15 for WST-8 assay. *p < 0.05, **p < 0.01, and ***p < 0.001, Tukey–Kramer HSD.

We evaluated the effect of the ROCK inhibitor Y-27632 on the TGF-β2-induced increase in TER and proliferation in SC cells. Y-27632 significantly decreased TER in SC cells (Fig. 1A). In addition, Y-27632 significantly suppressed the TGF-β2-induced elevation of TER in SC cells (Fig. 1A). By contrast, Y-27632 alone had no effect on cell proliferation but significantly inhibited TGF-β2-induced cell proliferation (Fig. 1B).

### Effect of a ROCK inhibitor on TGF-β2-induced changes in protein and mRNA expression

**Figure 2:**
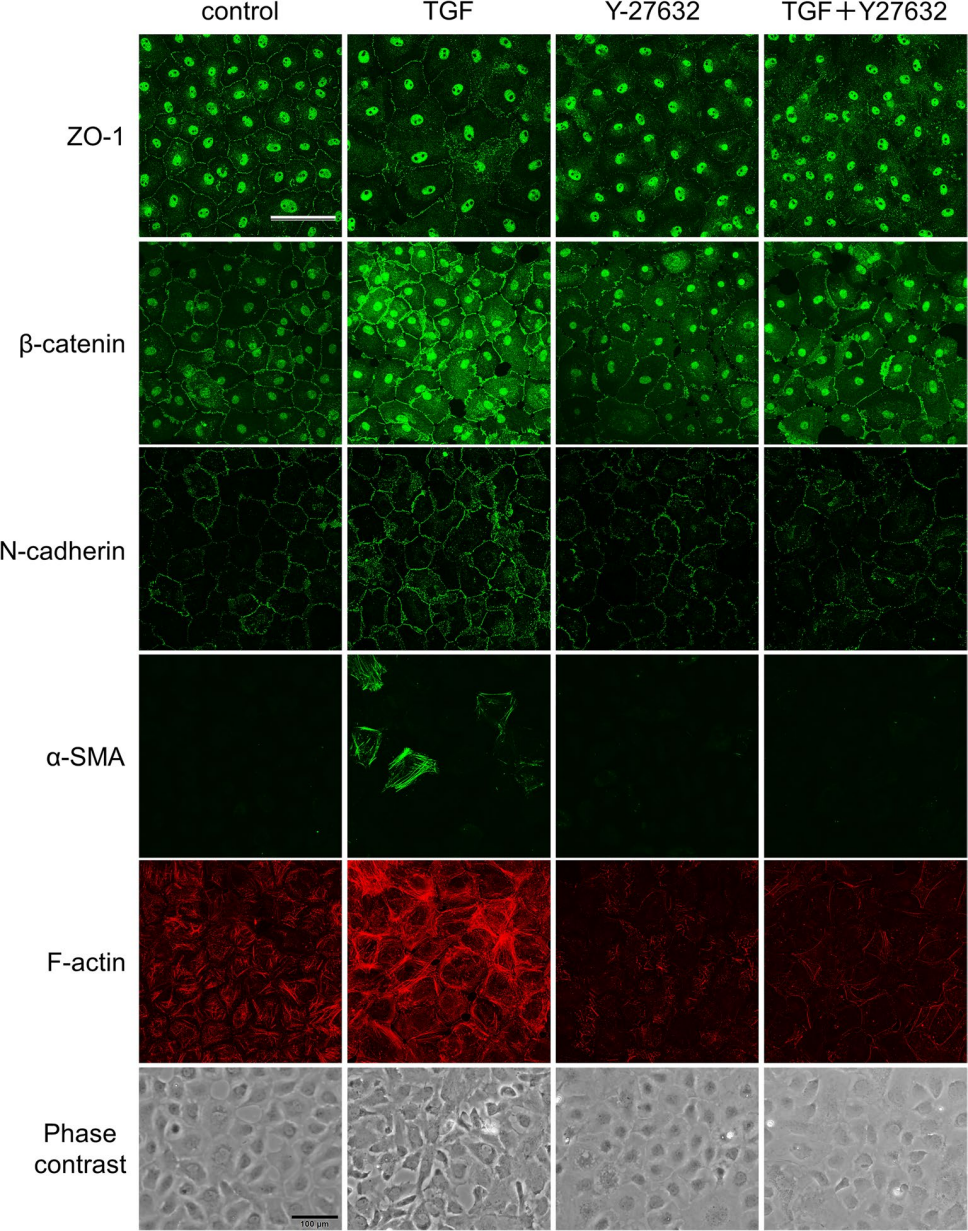
Immunohistochemistry of Schlemm’s canal (SC) cells treated with TGF-β2 and Y-27632. SC cells were treated with 5 ng/mL TGF-β2 and 10 µM Y-27632 for 72 h. ZO-1 (top, green), β-catenin (second, red), N-cadherin (third, green), α-smooth muscle actin (α-SMA; fourth, green), and F-actin (fifth, red) were visualized by immunostaining. Phase contrast images were shown at the bottom. Scale bar = 100 µm.

We performed immunostaining of cell–cell adhesion and cytoskeletal proteins in SC cells (Fig. 2). ZO-1, which forms tight junctions, did not show changes in expression upon TGF-β2 stimulation. Expression of N-cadherin and β-catenin increased near the cell membrane upon TGF-β2 stimulation, an effect suppressed by Y-27632. Cytoskeletal proteins such as F-actin and α-SMA were upregulated by TGF-β2 stimulation, and this effect was suppressed by Y-27632. In addition, Y-27632 inhibited TGF-β2-induced cell morphological changes.

**Figure 3:**
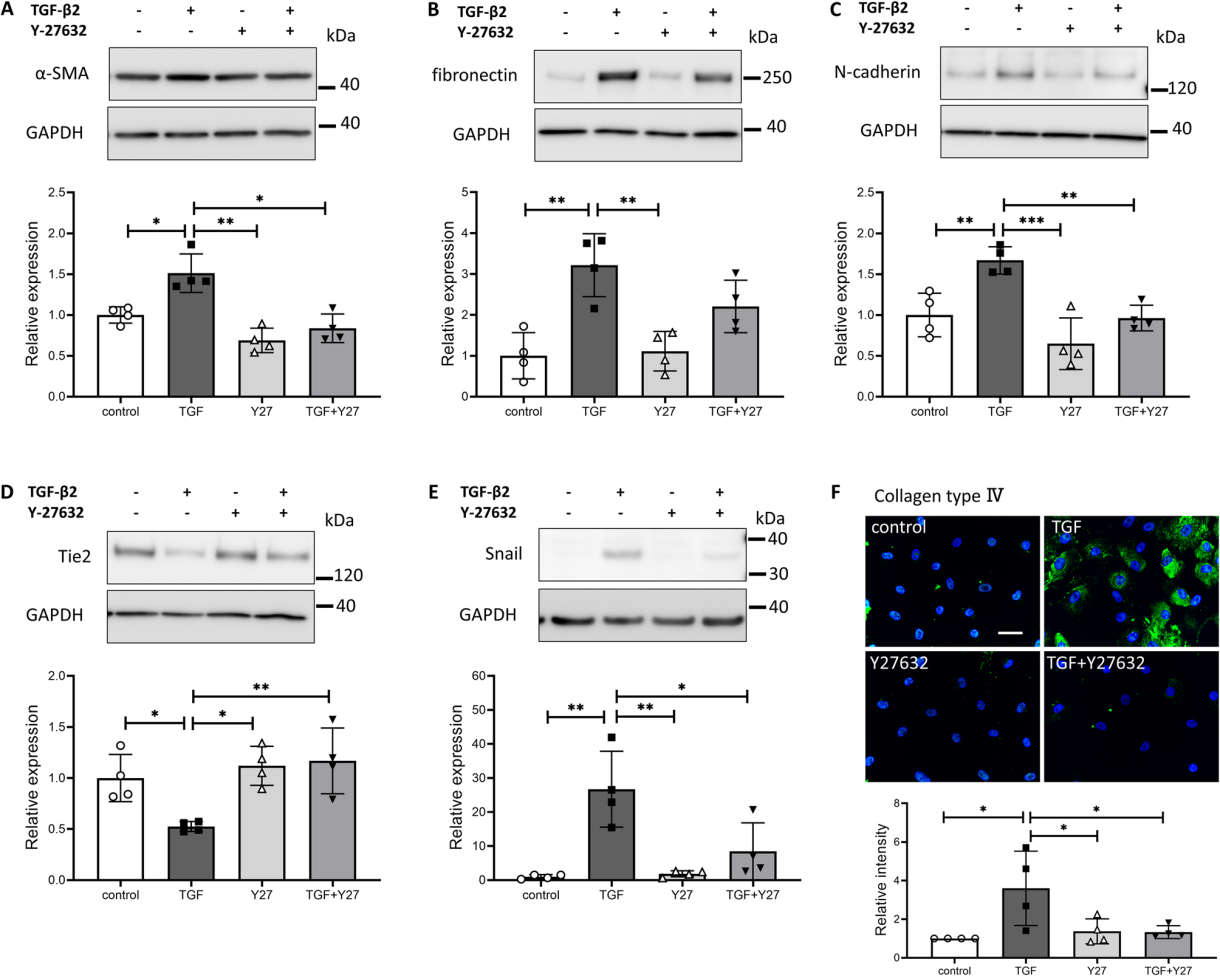
Effects of TGF-β2 and Y-27632 on protein levels in Schlemm’s canal (SC) cells. SC cells were treated with 5 ng/mL TGF-β2 and/or 10 µM Y-27632 for 72 h (A-D, F) or 24 h (E). α-SMA (**A**), fibronectin (**B**), N-cadherin (**C**), Tie2 (**D**), and Snail (**E**) levels evaluated by western blotting. (**F**) Collagen type IV level evaluated by immunohistochemistry (Scale bar = 50 µm). Data are means ± SD (n = 4). *p < 0.05, **p < 0.01, and ***p < 0.001, Tukey–Kramer HSD.

Western blotting was performed to compare the protein levels of α-SMA, fibronectin, and N-cadherin as mesenchymal markers, Tie2 as an endothelial marker, and Snail as an EndMT inducer. TGF-β2 significantly increased the levels of α-SMA, fibronectin, and N-cadherin at 72 h (Fig. 3A–C). Y-27632 significantly inhibited the TGF-β2-induced increase in the expression of α-SMA and N-cadherin. Moreover, Y-27632 partially inhibited the TGF-β2-induced increase in fibronectin expression, albeit not significantly so. Tie2 expression was significantly decreased by TGF-β2, an effect significantly suppressed by Y-27632 (Fig. 3D). The expression of Snail was significantly increased by TGF-β2 treatment for 24 h, and the effect was suppressed by Y-27632 (Fig. 3E). Furthermore, immunostaining showed that TGF-β2 increased the expression extracellular matrix collagen type IV, which was suppressed by the simultaneous addition of Y-27632 (Fig. 3F).

Real-time PCR showed that the expression of *ACTA2* (α-SMA), *CDH2* (N-cadherin), *SNAI1* (Snail), and *TEK* (Tie2) changed, in agreement with the western blotting results (Supplementary Fig. S1). In addition, bone morphogenic protein (BMP) 4, which has an endogenous inhibitory effect on TGF-β2, was significantly decreased by TGF-β2, and the effect was suppressed by Y-27632. Furthermore, levels of gremlin mRNA (*GREM1*), a BMP inhibitor, were increased by TGF-β2, and the effect was significantly suppressed by Y-27632.

### Effect of a ROCK inhibitor on canonical and non-canonical TGF-β2 signaling

**Figure 4:**
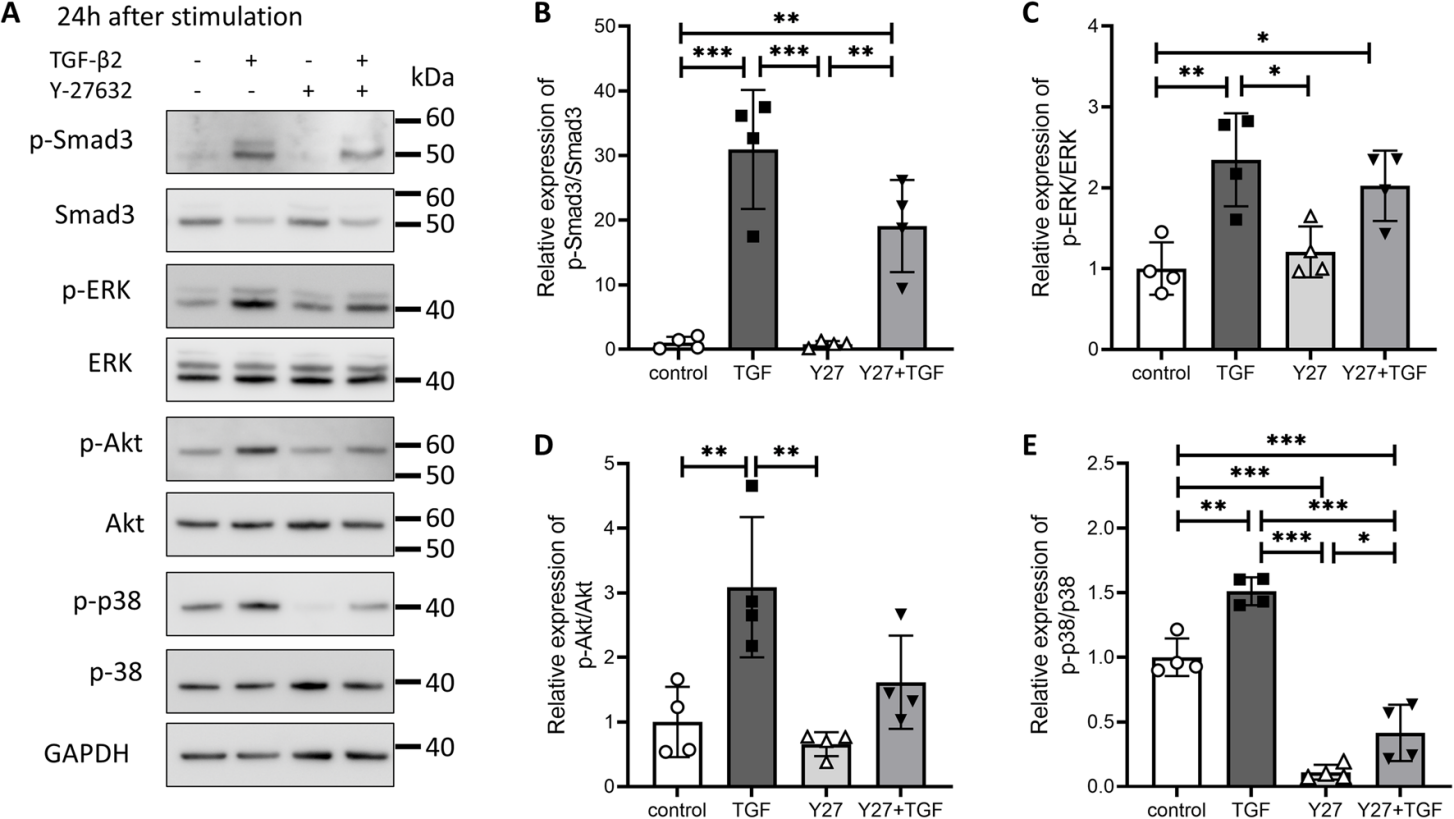
Effect of Y-27632 on TGF-β2-induced activation of the Smad and non-Smad pathways in Schlemm’s canal (SC) cells. SC cells were treated with TGF-β2 and Y-27632 for 24 h. (**A**) Representative phosphorylated and total bands of Smad3, ERK, Akt, and p38 proteins. Relative changes in phosphorylation levels of Smad3 (**B**), ERK (**C**), Akt (**D**), and p38 (**E**). Data are means ± SD (n = 4). *p < 0.05, **p < 0.01, and ***p < 0.001, Tukey–Kramer HSD.

Next, we investigated the effect of the ROCK inhibitor on TGF-β2-activated intracellular signals. TGF-β2-induced Smad3 phosphorylation (canonical signaling TGF-β2 pathway) was partially suppressed by Y-27632, albeit not significantly so (Fig. 4A, B). Phosphorylation of Akt, ERK, and p38 mitogen-activated protein kinase (MAPK) (non-canonical TGF-β2 pathways) was also investigated. Y-27632 had no effect on ERK phosphorylation (Fig. 4A, C). TGF-β2-induced Akt phosphorylation was suppressed, but not significantly so (Fig. 4A, D). Y-27632 most affected p38 MAPK phosphorylation, and TGF-β2-induced phosphorylation and Y-27632 alone suppressed baseline phosphorylation (Fig. 4A, E).

### Effect of BMP4 on TGF-β2-induced changes in SC cells

**Figure 5:**
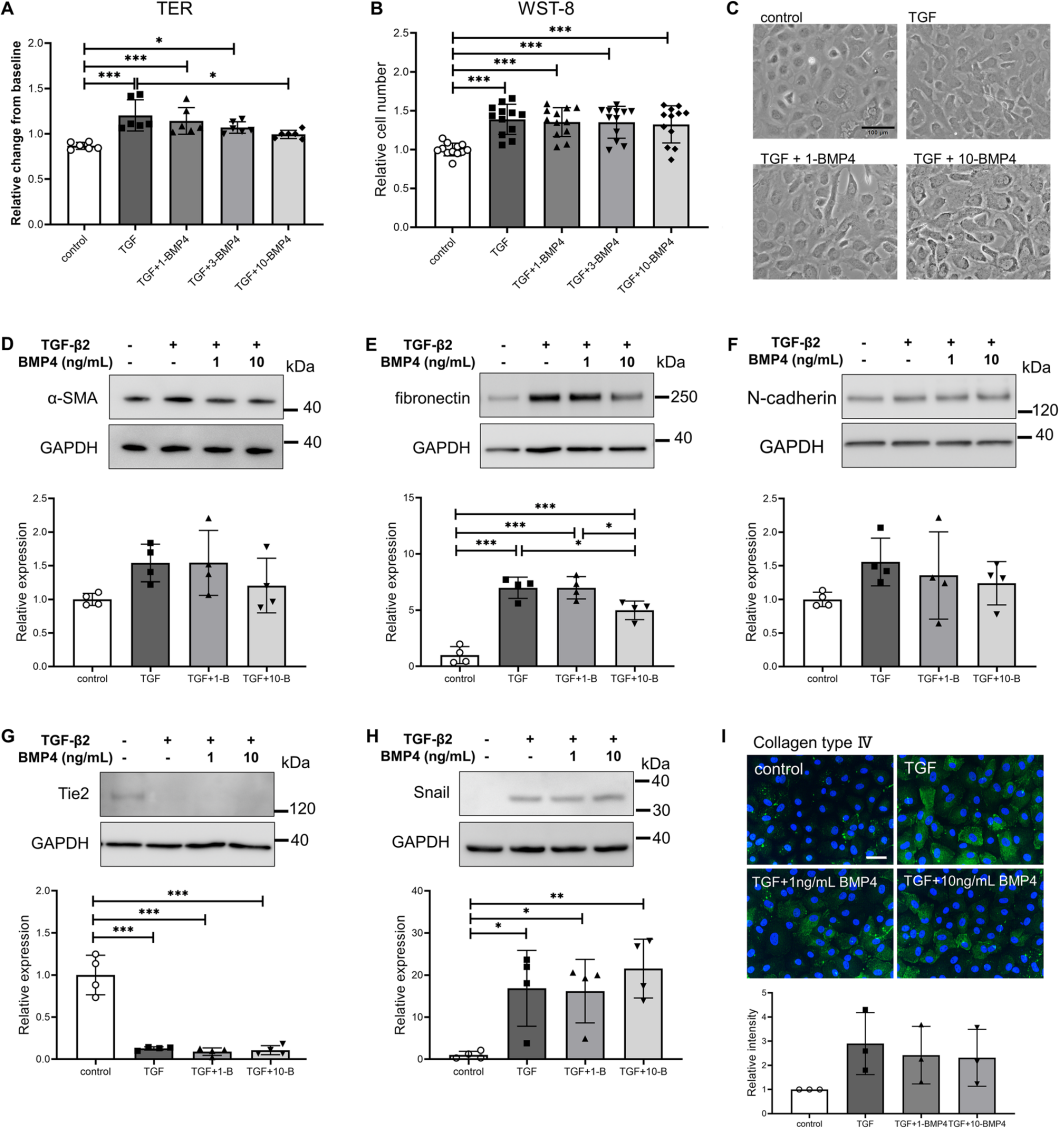
Effects of BMP4 on TGF-β2-induced changes in trans-endothelial electrical resistance (TER), cell proliferation, and protein levels in Schlemm’s canal (SC) cells. SC cells were treated with 5 ng/mL TGF-β2 and 1, 3, and 10 µM BMP4 for 72 h (**A**–**G**, **I**) or 24 h (**H**). (**A**) TER values as relative changes from baseline. (**B**) Proliferation of SC cells evaluated by WST-8 assay. (**C**) Phase-contrast images of SC cells (scale bar = 100 µm). α-SMA (**D**), fibronectin (E), N-cadherin (**F**), Tie2 (**G**), and Snail (**H**) levels evaluated by western blotting. (**I**) Collagen type IV level evaluated by immunohistochemistry (scale bar = 50 µm). Data are means ± SD; n = 6 (A); n = 12 (B); n = 4 (D-H); n = 3 (I). *p < 0.05, **p < 0.01, and ***p < 0.001, Tukey–Kramer HSD.

**Figure 6:**
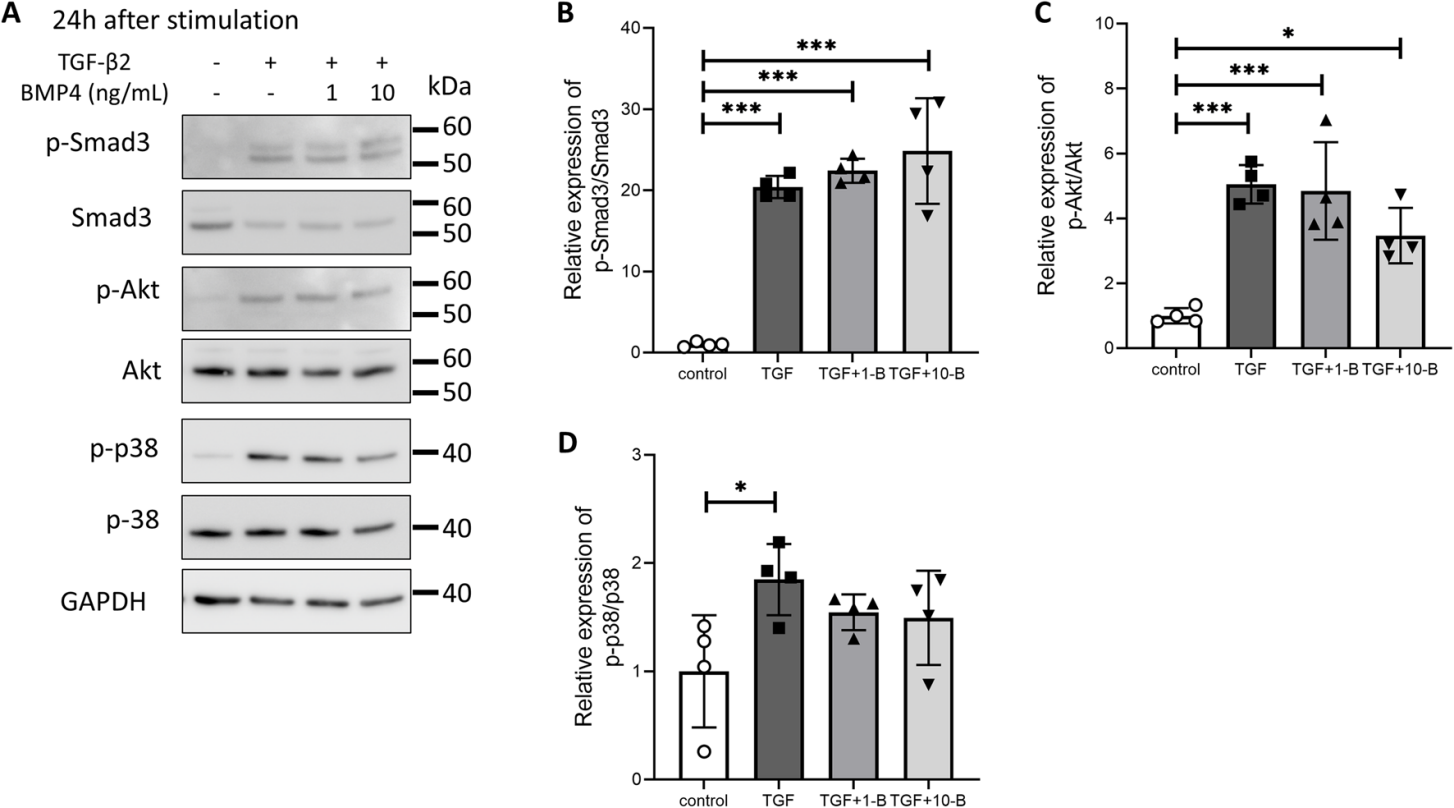
Effects of BMP4 on TGF-β2-induced activation of the Smad and non-Smad pathways in Schlemm’s canal (SC) cells. SC cells were treated with TGF-β2 and BMP4 for 24 h. (**A**) Representative phosphorylated and total bands of Smad3, Akt, and p38 proteins. Relative changes in phosphorylation levels of Smad3 (**B**), Akt (**C**), and p38 (**D**). Data are means ± SD (n = 4). *p < 0.05, **p < 0.01, and ***p < 0.001, Tukey–Kramer HSD.

Y-27632 inhibited both the TGF-β2-induced decrease in *BMP4* expression and the increase in *GREM1* expression. Therefore, we examined the effect of BMP4 on TGF-β2-induced EndMT of SC cells. Addition of 10 ng/mL BMP4 significantly suppressed the increase in TER caused by TGF-β2 (Fig. 5A). However, BMP4 did not suppress TGF-β2-induced cell proliferation and cell morphology change (Fig. 5B, C). BMP4 did not affect the TGF-β2-induced increase in α-SMA expression (Fig. 5D). Fibronectin expression was significantly suppressed by 10 ng/mL BMP4 (Fig. 5E). N-cadherin was slightly suppressed by BMP4, albeit not significantly so (Fig. 5F). BMP4 had no significant effect on the TGF-β2-induced changes in Tie2, Snail, and collagen type IV (Fig. 5G–I). BMP4 had no effect on TGF-β2-induced mRNA level changes (Supplementary Fig. S2). BMP4 had no effect on TGF-β2 intracellular signaling, such as Smad3, Akt, and p38 MAPK (Fig. 6).

### Effect of a p38 MAPK inhibitor on TGF-β2-induced changes in SC cells

**Figure 7:**
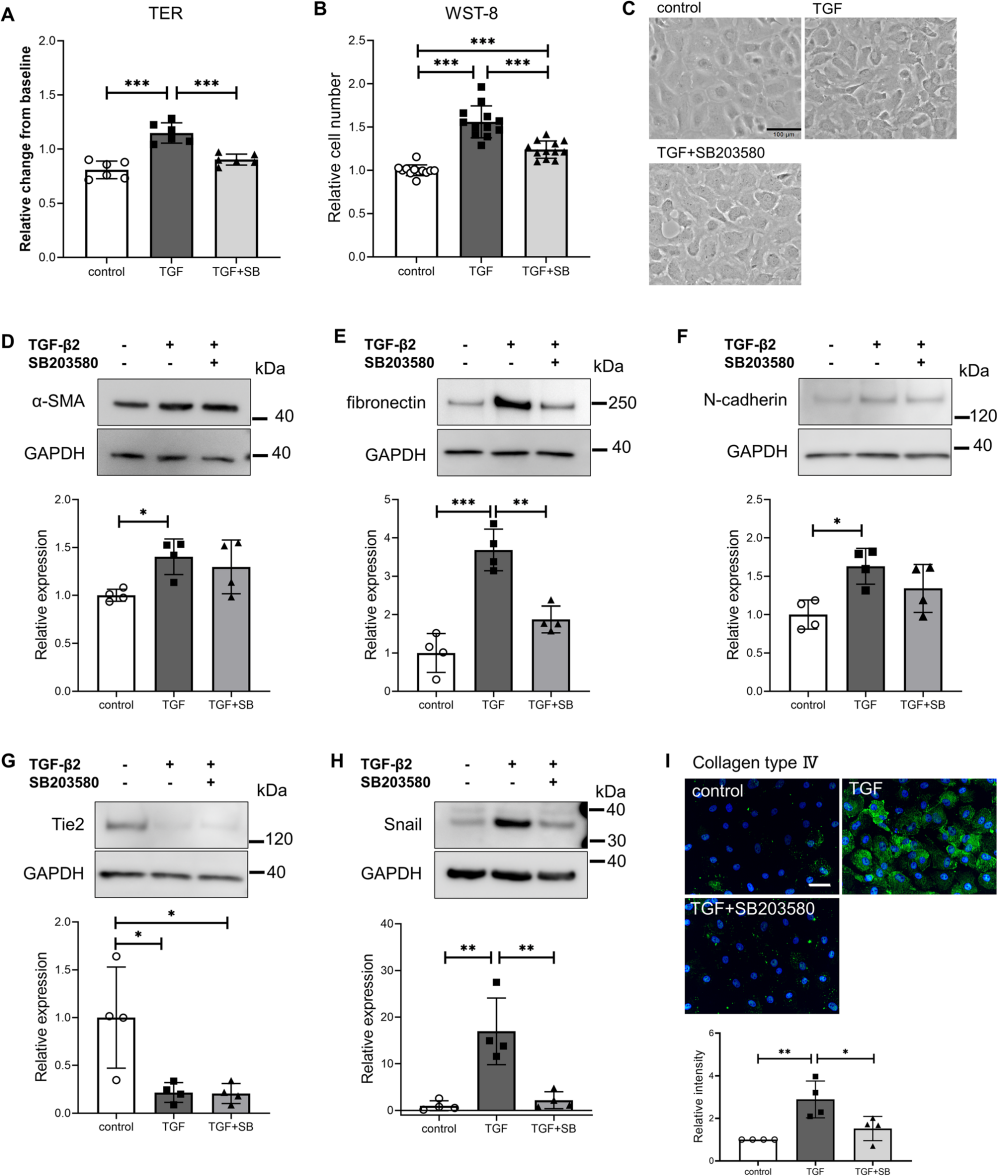
Effects of the p38 inhibitor SB203580 on the TGF-β2-induced changes in trans-endothelial electrical resistance (TER), cell proliferation, and protein levels in Schlemm’s canal **(SC) cells.** SC cells were treated with 5 ng/mL TGF-β2 and 10 µM SB203580 for 72 h (**A**–**G**, **I**) or 24 h (**H**). (**A**) TER values as relative changes from baseline. (**B**) Proliferation of SC cells evaluated by WST-8 assay. (**C**) Phase-contrast images of SC cells (scale bar = 100 µm). α-SMA (**D**), fibronectin (**E**), N-cadherin (**F**), Tie2 (**G**), and Snail (**H**) levels evaluated by western blotting. (**I**) Collagen type IV level evaluated by immunohistochemistry (scale bar = 50 µm). Data are means ± SD; n = 6 (A); n = 12 (B); n = 4 (D–I). *p < 0.05, **p < 0.01, and ***p < 0.001, Tukey–Kramer HSD.

Y-27632 significantly inhibited phosphorylation of p38 MAPK. We investigated the involvement of p38 MAPK in the EndMT induced by TGF-β2. SB203580, a p38 MAPK inhibitor, inhibited TGF-β2-induced TER elevation and cell proliferation (Fig. 7A, B). In addition, SB203580 tended to suppress cell morphological changes induced by TGF-β2 (Fig. 7C). SB203580 did not affect the increase in α-SMA expression (Fig. 7D). By contrast, SB203580 suppressed the elevation of fibronectin expression by TGF-β2 (Fig. 7E). N-cadherin was slightly inhibited by SB203580, but not significantly so (Fig. 7F). SB203580 had no effect on the TGF-β2-induced decrease in Tie2 (Fig. 7G). The increases in Snail and collagen type IV expression mediated by TGF-β2 were significantly inhibited by SB203580 (Fig. 7H, I). Real-time PCR showed that SB203580 caused changes in the expression of *ACTA2*, *CDH2*, *SNAI1*, and *TEK*, in agreement with the western blotting results (Supplementary Fig. S3). SB203580 had no effect on *BMP4* expression but significantly suppressed *GREM1* expression (Supplementary Fig. S3).

## Discussion

SC cells from glaucoma patients show increased expression of the fibrosis markers α-SMA, fibronectin, and collagen, and increased cell proliferation^19,20^. These changes are similar to those induced by TGF-β2 stimulation^17,18^. Our findings imply induction of EndMT by TGF-β2 in SC cells. Therefore, EndMT may be induced by aqueous humor cytokines such as TGF-β2 in the SC of glaucoma patients, resulting in decreased endothelial function and decreased aqueous humor outflow control. Furthermore, Y-27632 suppressed the TGF-β2-induced EndMT in SC cells. Suppression of EMT or EndMT by ROCK inhibitors has been reported in corneal endothelial cells, retinal pigment epithelial cells, lens epithelial cells, and lung epithelial cells^31–35^. These results indicate that ROCK inhibitors act directly on SC cells in glaucoma patients, suppress the EndMT induced by TGF-β2 in the aqueous humor, and restore endothelial function to normal, thereby improving outflow resistance. However, because Y-27632 does not suppress the decrease in *PECAM1* expression and the increase in *FN1* and *COL4A1* expression induced by TGF-β2 (Supplementary Fig. S1), ROCK inhibitors do not completely restore endothelial function. In addition, we did not examine the mesenchymal-to-endothelial transition, the reverse of EndMT, which is a subject for future investigation.

BMP4 reportedly affects intraocular pressure, and expression of gremlin, which acts as its endogenous inhibitor, causes intraocular pressure elevation^36,37^. In this study, TGF-β2 stimulation decreased the expression of *BMP4* and increased that of *GREM1*. In addition, a ROCK inhibitor significantly suppressed TGF-β2-induced *BMP4* downregulation and *GREM1* upregulation. Furthermore, BMP4 stimulation significantly suppressed the fibronectin expression and TER elevation induced by TGF-β2. BMP4 reportedly suppresses EMT in lens epithelial cells and retinal pigment epithelial cells^38,39^. These results implicate the suppression of fibronectin expression in SC cells via BMP4 signaling in the improvement of aqueous outflow resistance by ROCK inhibitors. In addition, the effect of BMP4 in the presence of TGF-β2 was observed only at a high concentration (10 ng/mL), and BMP4 had no effect on TGF-β2-induced mRNA level changes (Supplementary Fig. S2). These results are thought to be related to the TGF-β2-induced increase in gremlin expression.

Y-27632 significantly inhibited p38 phosphorylation irrespective of the presence or absence of TGF-β2. Activation of Rho/ROCK upstream of p38 MAPK has been reported in orbital fibroblasts and microglia^40,41^. Therefore, p38 MAPK is likely located downstream of the Rho/ROCK signal in SC cells. SB203580, a p38 MAPK inhibitor, significantly suppressed TGF-β2-induced upregulation of fibronectin, collagen type IV, and Snail, implying that p38 MAPK is an important signal in TGF-β2-induced EndMT induction. However, unlike the Y-27632, p38 MAPK inhibition had no significant effect on TGF-β2-induced Tie2 expression reduction and N-cadherin expression elevation. In this study, although there was no statistically significant difference, Y-27632 showed a tendency to suppress the phosphorylation of Akt and Smad3 by TGF-β2. It has been reported in other endothelial cells that suppression of Akt^42,43^ and Smad^44–46^ signals suppress EndMT. In addition, we previously reported that HDAC inhibitors significantly suppressed Akt signaling and suppressed EndMT in SC cells^17^. These reports suggest that the inhibition of Akt and Smad3 phosphorylation by ROCK inhibitors also contributes to the suppression of EndMT along with the suppression of p38 MAPK signals.

In conclusion, the ROCK inhibitor Y-27632 suppressed the TGF-β2-induced EndMT in SC cells. The effect may be related to inhibition of p38 MAPK and activation of BMP4 signal transduction.

## Experimental procedures

### Cell culture

Primary monkey SC cells were isolated from enucleated eyes of cynomolgus monkeys as described previously^17,29^. The cells were cultured in low-glucose Dulbecco’s modified Eagle’s medium (DMEM; 041-29775, FUJIFILM Wako Pure Chemical, Osaka, Japan) in the presence of 10% FBS (SH30910.03, HyClone™, Cytiva, Tokyo, Japan), glutamine (2 mM), penicillin (100 U/mL), streptomycin (100 μg/mL, 10378016, Penicilin-Streptomycin-glutamine, Thermo Fisher Scientific, Rockford, IL), and amphotericin B (0.5 μg/mL, 15290018, Thermo Fisher Scientific) at 37 °C in 5% CO_2_. Cells were used after three to five passages.

### Measurement of trans-endothelial electrical resistance

TER was measured as described previously^47^. SC cells were seeded on Transwell polyester membrane insert (0.4 μm pore size, 6.5 mm diameter; 3470, Corning Inc., Corning, NY) in 24-well culture plates and cultured for 7 days or more until the TER values were stabilized. The cells were cultured in serum-free DMEM overnight before treatment. Then they were treated with 5 ng/mL human recombinant TGF-β2 (302-B2, R&D Systems, Minneapolis, MN) with or without Y-27632 (259-00613, FUJIFILM Wako Pure Chemical), human recombinant bone morphogenic protein 4 (BMP4, 022-17071, FUJIFILM Wako Pure Chemical) or SB203580 (S1076, Selleck Chemicals, Huston, TX). TER was measured at 24, 48, and 72 h after treatment. At least three independent experiments were performed.

### Cell proliferation assay

Cell proliferation was evaluated using the WST-8 assay (Cell Counting Kit-8, CCK-8; CK04, Dojindo, Kumamoto, Japan), as described previously^17^. SC cells were seeded in 96-well plates at 1 × 10^4^ cells per well and incubated for 24 h. After serum starvation for 24 h, TGF-β2, Y-27632, BMP4 and SB203580 were added to the cells and incubated for 72 h. CCK-8, which is a detection reagent, was added and the absorbance at 450 nm was measured using a microplate reader (Multiskan FC, Thermo Fisher Scientific) after incubation for 2 h. Cell proliferation is presented as relative change compared to the control.

### Immunocytochemistry

Fluorescent immunostaining of SC cells was performed as reported previously^17,47^. Phase-contrast images were acquired with an inverted microscope (IX71, Olympus, Tokyo, Japan) before fixation. Treated cells were fixed with 4% (v/v) paraformaldehyde in PBS for 15 min at room temperature. After washing with cytoskeletal buffer (10 mM 2-morpholinoethanesulfonic acid potassium salt, 150 mM NaCl, 5 mM EGTA, 5 mM MgCl_2_, and 5 mM glucose, pH 6.1), the cells were treated with 0.5% (v/v) Triton X-100 in PBS for 12 min at room temperature. For blocking, the cells were treated with serum buffer (10% FBS and 0.2 mg/mL sodium azide in PBS) at 4 °C for at least 2 h. The cells were incubated with primary antibodies (see Table 1) overnight at 4 °C. Next, the cells were incubated with an anti-mouse or anti-rabbit IgG secondary antibody labeled with Alexa Fluor 488 at room temperature for 30 min. For visualization of F-actin, phalloidin labeled with Alexa Fluor 546 was incubated with the secondary antibody. After mounting with VECTASHIELD mounting medium containing 4,’ 6-diamidino-2-phenylindole (DAPI; H-1200, Vector Laboratories, Burlingame, CA), the cells were observed under a laser confocal microscope (FV-1200; Olympus) or an all-in-one epifluorescence microscope (BZ-X710; Keyence, Itasca, IL, USA). We performed immunostaining in at least three independent experiments.

**Table 1:** Antibodies for immunocytochemistry.

Target protein	Catalog number	Dilution	Source
Collagen type IV	GTX26586	1/100	GeneTex Inc, Irvine, CA
α-SMA	19245	1/200	Cell Signaling Technology, Danvers, MA
N-cadherin	14215	1/200	
β-catenin	C2206	1/1000	Sigma-Aldrich, Merck KGaA, Dermstadt, Germany
ZO-1	617300	1/100	Invotrogen, Thermo Fisher Scientific, Rockford, IL, USA
Alexa Fluor™ 488 goat anti-mouse IgG (H + L)	A11001	1/1000	
Alexa Fluor™ 488 goat anti-rabbit IgG (H + L)	A11008	1/1000	
Alexa Fluor™ 546 phalloidin	A22283	1/200	

### Western blotting

Western blotting was performed as described previously^17,30^. Cell lysates were collected from SC cells 24 and 72 h after treatment using LIPA buffer (89900, Thermo Fisher Scientific) with protease inhibitor (78410, Thermo Fisher Scientific) and phosphatase inhibitor (07574-61, Nacarai Tesque, Kyoto, Japan). Loading samples were prepared from the cell lysates with NuPAGE LDS sample buffer and dithiothreitol (Thermo Fisher Scientific). Samples were loaded onto a NuPAGE Bis–Tris or Tris–acetate polyacrylamide gel (Thermo Fisher Scientific), and proteins were separated by sodium dodecyl sulfate–polyacrylamide gel electrophoresis. These proteins were transferred onto polyvinylidene difluoride membranes by electroblotting. The membranes were blocked with 2% bovine serum albumin (BSA; 018-15154, FUJIFILM Wako Pure Chemical), or 5% skim milk (31149-75, Nacalai Tesque) in Tris-buffered saline containing 0.1% Tween-20 (TBS-T) for 1 h at room temperature. The membranes were incubated with primary antibodies (Table 2) diluted with 5% BSA or 5% skim milk in TBS-T overnight at 4 °C. After washing with TBS-T, the membranes were incubated with horseradish peroxidase (HRP)-conjugated secondary antibodies for 30 min at room temperature. The chemiluminescence signal was detected using ECL Prime (RPN2232, Cytiva) or ECL Select western blotting detection reagent (PRN2235, Cytiva) and a luminescence imager (LAS 4000mini; FUJIFILM, Tokyo, Japan). All membranes were stripped of antibodies using WB stripping solution (05364-55, Nacalai Tesque) and incubated with an anti-glyceraldehyde-3-phosphate dehydrogenase (GAPDH) antibody followed by an HRP-conjugated rabbit IgG antibody as a loading control. The densitometry of immunoreactive bands was analyzed using Image J software (National Institutes of Health, Bethesda, MD).

**Table 2:** Antibodies for Western blot analysis.

Target protein	Catalog number	Dilution	Source
Fibronectin	ab6328	1/2000	Abcam, Cambridge, UK
Smad3	Ab40854	1/2000	
p-Smad3(S423/425)	ab52903	1/2000	
α-SMA	19245	1/2000	Cell Signaling Technology, Danvers, MA
N-cadherin	4061	1/1000	
Snail	3879	1/1000	
Tie2	7403	1/1000	
p38	9212	1/1000	
p-p38 (T180/Y182)	4631	1/1000	
ERK1/2	9102	1/1000	
p-ERK1/2 (T202/Y204)	9101	1/1000	
Akt	9272	1/1000	
p-Akt (S473)	9271	1/1000	
Anti-rabbit IgG HRP	7074	1/2000	
Anti-mouse-IgG HRP	7076	1/2000-5000	
GAPDH	G8795	1/10000	Sigma-Aldrich, Merck KGaA, Darmstadt, Germany

### Real time RT-PCR

Real time RT-PCR was performed as described previously^17,48^. RNA samples were prepared from SC cells using a NucleoSpin RNA Kit (U0955, Takara Bio, Shiga, Japan), according to the manufacturer’s instructions. The concentration of RNA samples was measured using a DS-11 NanoPad spectrophotometer (DeNovix, Wilmington, DE). Reverse transcription was performed using PrimeScript™ RT Master Mix (RR036A, Takara Bio) according to the manufacturer’s protocol. Quantitative PCR was performed using TB Green® EX Taq II (RR820A, Takara Bio) and the StepOnePlus Real-Time PCR System (Thermo Fisher Scientific). Relative expression of target mRNAs was compared to the control samples using the comparative threshold cycle method; GAPDH was used as an endogenous control based on the results of preliminary studies (Supplementary Fig. S4). The primer sequences are listed in Table 3.

**Table 3:** Primer sequences for monkey genes examined by quantitative RT-PCR.

Gene	Primer sequences (5′ to 3′)	Product size (bp)
*ACTA2*	F: CCAGCCAAGCACTGTCAGGAATC	114
	R: AGCAAAGCCGGCCTTACAGA	
*CDH2*	F: GACGCAGCTCGGAAGTGTCT	123
	R: AAGCCTCTACAGACGCCCCT	
*SNAI1*	F: GTCCGGCCTTGTGAGTGGTA	169
	R: GCTGCTGGAAGGTAAACTCTGG	
*TEK*	F: ATAGGGTCAAGCAACCCAGCC	70
	R: GGTCCGCTGGTGCTTCAGAT	
*BMP4*	F: GGAGCTTCCACCACGAAGAACA	172
	R: AAGCCCCGTTCCCAATCAGG	
*GREM1*	F: AGCCGCATTGACAGTATGAGC	113
	R: ATGGCACCTTGGGACCCTTTC	
*PECAM1*	F: ACACGGAAGTGCAAGTGTCCT	136
	R: AGGGAGCCTTCCGTCCTAGAG	
*FN1*	F: ACAAGCGTGTCTCTCTGCC	149
	R: CCAGGGTGATGCTTGGAGAA	
*COL4A1*	F: AGGTCGCCCGGGATTTAATG	149
	R: GTACCCCAATGCTCCCCTTC	
*GAPDH*	F: TCGTCATCAATGGAAGCCCC	136
	R: AAATGAGCCCCAGCCTTCTC	
*ACTB*	F: CAGCTCGCCATGGATGATGATA	111
	R: GATGGAGGGGAAGACGGCTC	
*18S rRNA*	F: GCCCGAAGCGTTTACTTTGA	93
	R: CCGCGGTCCTATTCCATTATT	

ACTA2, Actin alpha 2 (smooth muscle); CDH2, Cadherin 2 (N-cadherin); SNAI1, Snai family transcriptional repressor 1 (Snail); TEK, TEK Receptor tyrosine kinase (Tie2); BMP4, Bone morphogenic protein 4; GREM1, Gremlin 1; PECAM1, Platelet and endothelial cell adhesion molecule 1 (CD31); FN1, fibronectin 1; COL4A1, Collagen type IV alpha 1 chain; GAPDH, Glyceraldehyde 3-phosphate dehydrogenase; ACTB, Actin beta; 18S rRNA, 18S ribosomal RNA.

### Statistical analysis

Data are shown as means ± standard deviation (SD). JMP statistical software (version 14.3.0; SAS Institute, Cary, NC) was used for statistical analysis. Comparison of multiple groups was conducted using the Tukey–Kramer honestly significant difference (HSD) test. In all analyses, differences were considered statistically significant at *p* < 0.05.

## Supplementary Information

Supplementary Figures.

## Data Availability

All data generated or analyzed during this study are included in this published article and its Supplementary Information files.

## Abbreviations

TM: Trabecular meshwork
SC: Schlemm’s canal
ECM: Extra cellular matrix
TGF-β2: Transforming growth factor-beta 2
POAG: Primary open angle glaucoma
α-SMA: Alpha smooth muscle actin
EMT: Epithelial–mesenchymal transition
EndMT: Endothelial–mesenchymal transition
TER: Trans-endothelial electrical resistance
BMP: Bone morphogenetic protein
MAPK: Mitogen-activated protein kinase
