# Supplementary material for: A ROCK inhibitor suppresses the transforming growth factor-beta-2-induced endothelial–mesenchymal transition in Schlemm’s canal endothelial cells
Source: Sci Rep. 2023 Jun 14;13:9655. doi: 10.1038/s41598-023-36808-8 (PMC10267201; doi:10.1038/s41598-023-36808-8)
Supplement: Supplementary file 1 — Supplementary Figures. [file 41598_2023_36808_MOESM1_ESM.docx]

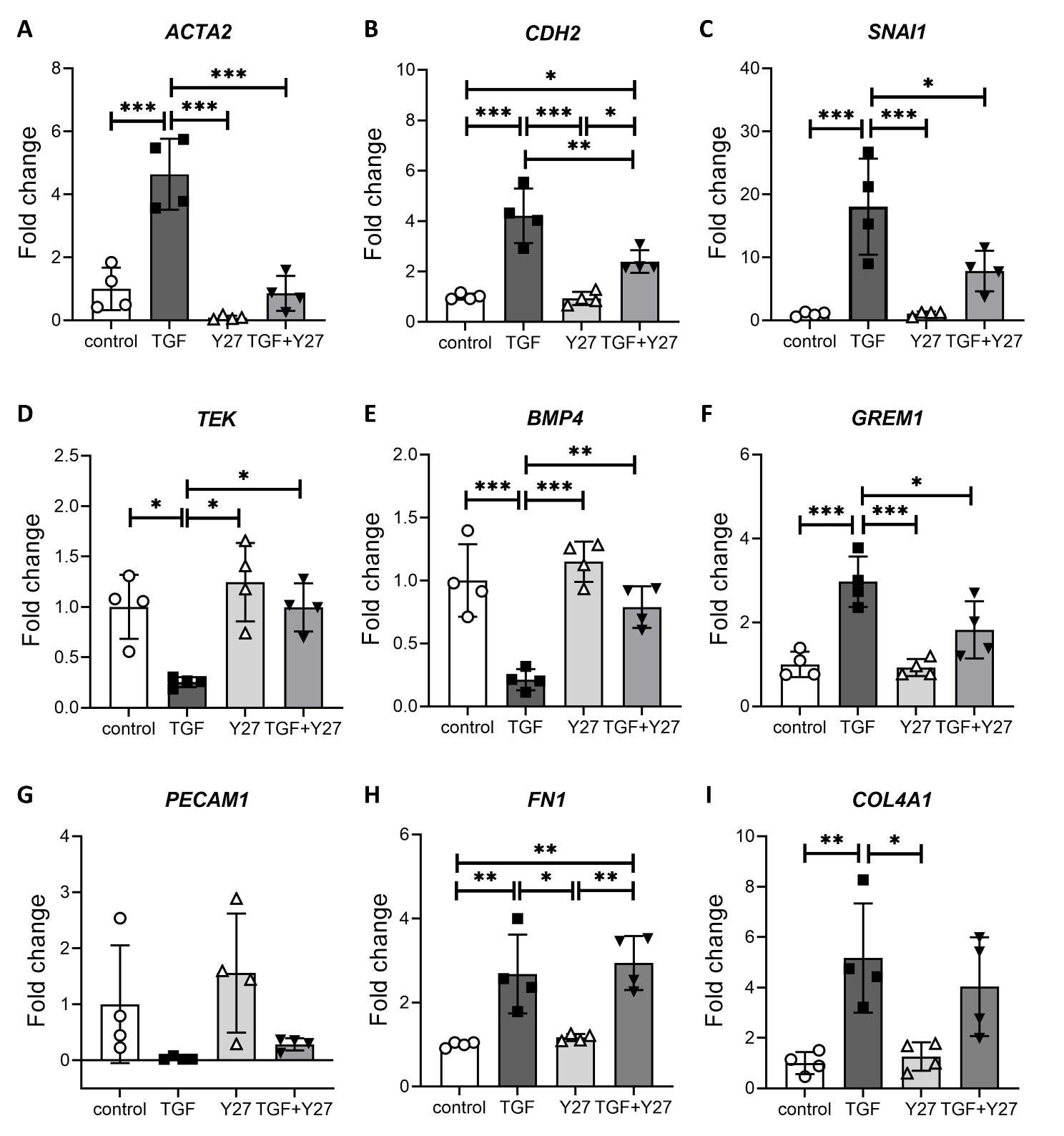


**Supplementary Figure S1. Effects of TGF-β2 and Y-27632 on mRNA levels in Schlemm’s canal (SC) cells.** SC cells were treated with 5 ng/mL TGF-β2 and/or 10 µM Y-27632 for 24 h. The mRNA levels of *ACTA2* (α-SMA; A), *CDH2* (N-cadherin; B), *SNAI1* (Snail; C), *TEK* (Tie2; D), *BMP4* (E), *GREM1* (gremlin; F), *PECAM1* (G), *FN1* (fibronectin; H), and *COL4A1* (collagen type4 alpha1; I) were evaluated by real-time RT-PCR. Data are means ± SD (n = 4). **p* < 0.05, ***p* < 0.01, and ****p* < 0.001, Tukey–Kramer HSD.


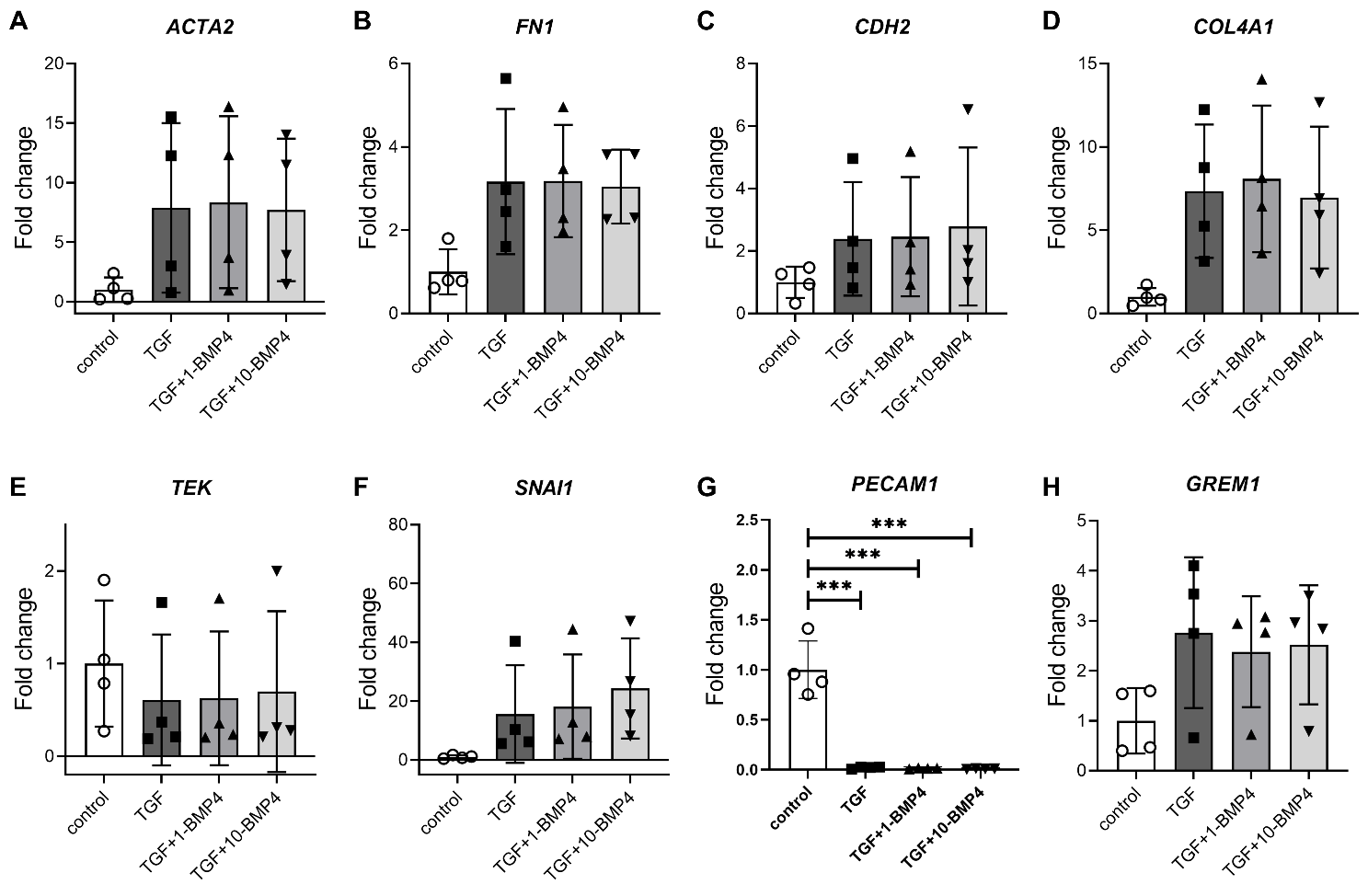


**Supplementary Figure S2. Effect of BMP4 on TGF-β2 induced mRNA level changes in Schlemm’s canal (SC) cells.** SC cells were treated with 5 ng/mL TGF-β2 and 1 or 10 ng/mL BMP4 for 24 h. The mRNA levels of *ACTA2* (α-SMA; A), *FN1* (fibronectin; B), *CDH2* (N-cadherin; C), *COL4A1* (collagen type4 alpha1; D), *TEK* (Tie2; E), *SNAI1* (Snail; F), *PECAM1* (G), and *GREM1* (gremlin; H) were evaluated by real-time RT-PCR. Data are means ± SD (n = 4). ****p* < 0.001, Tukey–Kramer HSD.


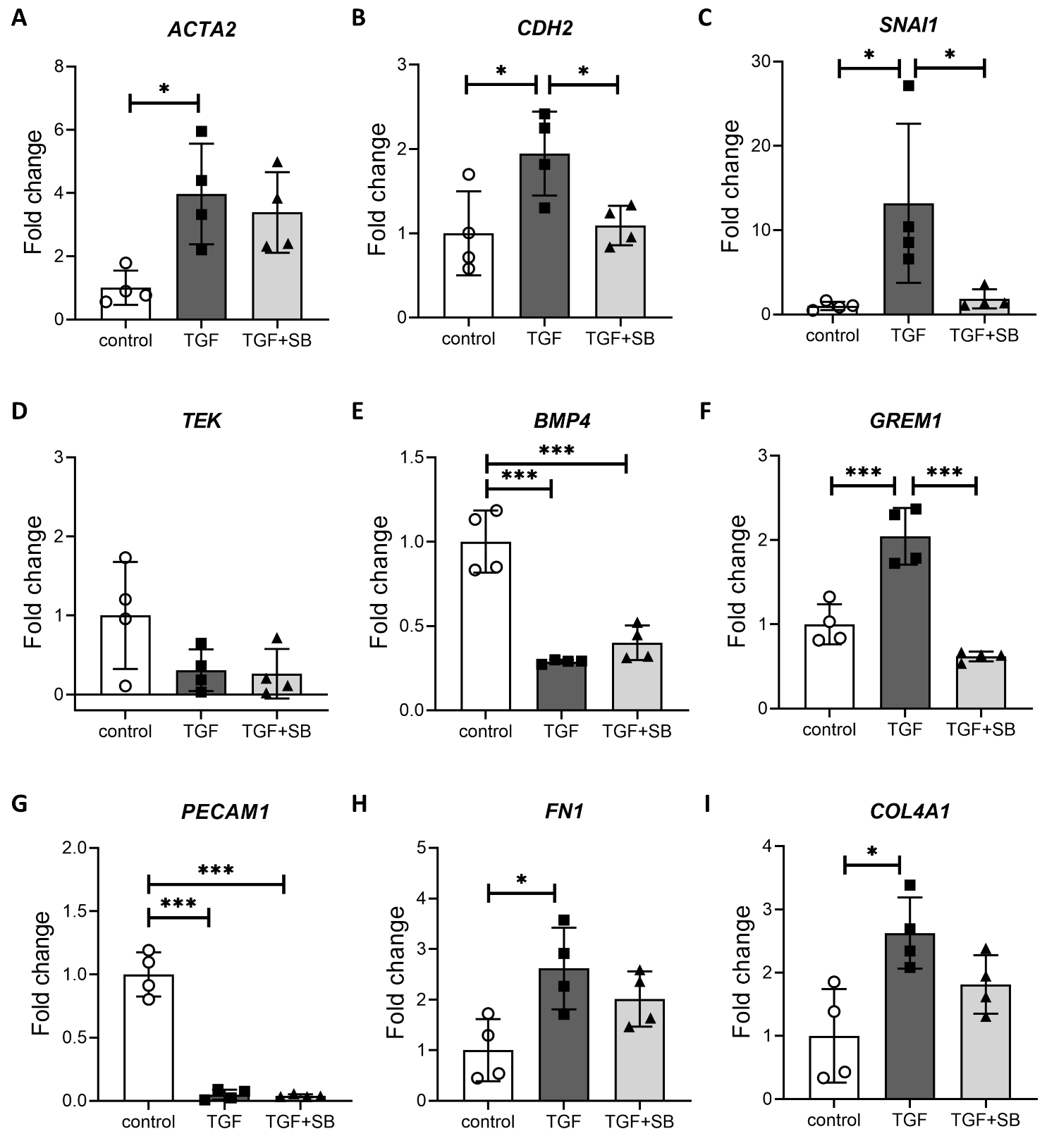


**Supplementary Figure S3. Effect of SB203580 on TGF-β2 induced mRNA level changes in Schlemm’s canal (SC) cells.** SC cells were treated with 5 ng/mL TGF-β2 and 10 µM SB203580 for 24 h. The mRNA levels of *ACTA2* (α-SMA; A), *CDH2* (N-cadherin; B), *SNAI1* (Snail; C), *TEK* (Tie2; D), *BMP4* (E), *GREM1* (gremlin; F), *PECAM1* (G), *FN1* (fibronectin; H), and *COL4A1* (collagen type4 alpha1; I) were evaluated by real-time RT-PCR. Data are means ± SD (n = 4). **p* < 0.05, ***p* < 0.01, and ****p* < 0.001, Tukey–Kramer HSD.


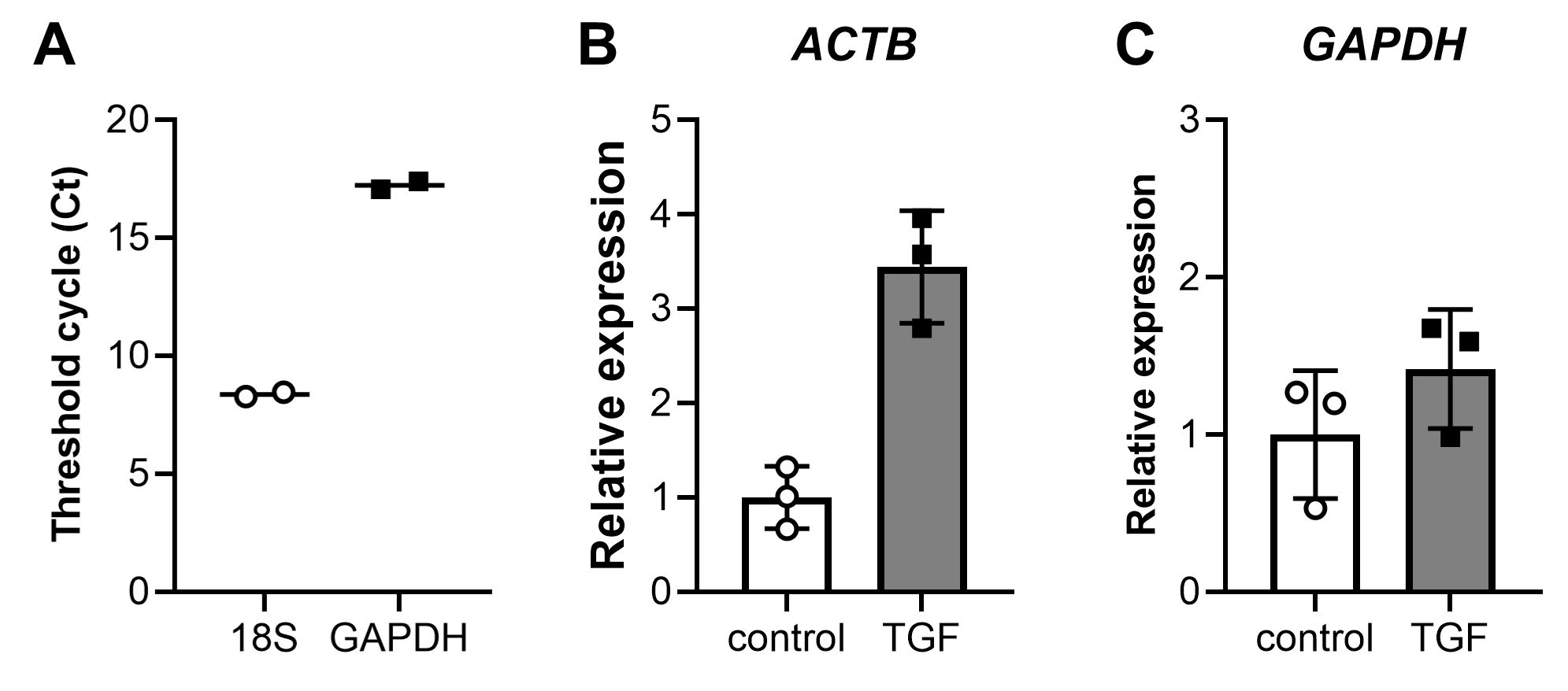


**Supplementary Figure S4. Preliminary qPCR data of house-keeping gene.** (A) Threshold cycles (Ct) of 18S ribosomal RNA and GAPDH are shown (n=2). (B, C) 5 ng/mL TGF-β2 for 24 h. The mRNA levels of *ACTB* (beta-actin, B) and *GAPDH* (C). Data are means ± SD (n = 3).
